# Bioelectronic modulation of carotid sinus nerve activity in the rat: a potential therapeutic approach for type 2 diabetes

**DOI:** 10.1007/s00125-017-4533-7

**Published:** 2018-01-14

**Authors:** Joana F. Sacramento, Daniel J. Chew, Bernardete F. Melo, Matteo Donegá, Wesley Dopson, Maria P. Guarino, Alison Robinson, Jesus Prieto-Lloret, Sonal Patel, Bradley J. Holinski, Nishan Ramnarain, Victor Pikov, Kristoffer Famm, Silvia V. Conde

**Affiliations:** 1 CEDOC, NOVA Medical School, Faculdade de Ciências, Universidade NOVA de Lisboa, Rua Camara Pestana, no. 6, 6A, edificio II, piso 3, 1150-082 Lisboa, Portugal; 2 Galvani Bioelectronics, Stevenage, UK; 3 Escola Superior de Saúde de Leiria-Instituto Politécnico de Leiria, Leiria, Portugal; 4 GlaxoSmithKline, Stevenage, UK

**Keywords:** Carotid body, Carotid sinus nerve, Glucose tolerance, Insulin resistance, KHFAC modulation, Neuromodulation, Type 2 diabetes

## Abstract

**Aims/hypothesis:**

A new class of treatments termed bioelectronic medicines are now emerging that aim to target individual nerve fibres or specific brain circuits in pathological conditions to repair lost function and reinstate a healthy balance. Carotid sinus nerve (CSN) denervation has been shown to improve glucose homeostasis in insulin-resistant and glucose-intolerant rats; however, these positive effects from surgery appear to diminish over time and are heavily caveated by the severe adverse effects associated with permanent loss of chemosensory function. Herein we characterise the ability of a novel bioelectronic application, classified as kilohertz frequency alternating current (KHFAC) modulation, to suppress neural signals within the CSN of rodents.

**Methods:**

Rats were fed either a chow or high-fat/high-sucrose (HFHSu) diet (60% lipid-rich diet plus 35% sucrose drinking water) over 14 weeks. Neural interfaces were bilaterally implanted in the CSNs and attached to an external pulse generator. The rats were then randomised to KHFAC or sham modulation groups. KHFAC modulation variables were defined acutely by respiratory and cardiac responses to hypoxia (10% O_2_ + 90% N_2_). Insulin sensitivity was evaluated periodically through an ITT and glucose tolerance by an OGTT.

**Results:**

KHFAC modulation of the CSN, applied over 9 weeks, restored insulin sensitivity (constant of the insulin tolerance test [K_ITT_] HFHSu sham, 2.56 ± 0.41% glucose/min; K_ITT_ HFHSu KHFAC, 5.01 ± 0.52% glucose/min) and glucose tolerance (AUC HFHSu sham, 1278 ± 20.36 mmol/l × min; AUC HFHSu KHFAC, 1054.15 ± 62.64 mmol/l × min) in rat models of type 2 diabetes. Upon cessation of KHFAC, insulin resistance and glucose intolerance returned to normal values within 5 weeks.

**Conclusions/interpretation:**

KHFAC modulation of the CSN improves metabolic control in rat models of type 2 diabetes. These positive outcomes have significant translational potential as a novel therapeutic modality for the purpose of treating metabolic diseases in humans.

**Electronic supplementary material:**

The online version of this article (10.1007/s00125-017-4533-7) contains peer-reviewed but unedited supplementary material, which is available to authorised users.

## 

## Introduction

The principal defects in type 2 diabetes are peripheral insulin resistance, abnormal hepatic glucose metabolism and progressive pancreatic beta cell failure. Glucose control in type 2 diabetes deteriorates progressively over time and, after failure of diet and exercise alone, needs on average a new intervention with glucose-lowering agents every 3–4 years in order to obtain/retain good control. Despite combination therapy and/or insulin treatment, a sizeable proportion of individuals remain poorly controlled [1]. The global burden of disease continues to increase and the number of individuals with type 2 diabetes will exceed 500 million by 2040 [2, 3]. There remains a need for novel therapeutic approaches with different mechanistics. We have previously shown that the abolition of carotid body activity through surgical resection of its sensitive nerve, the carotid sinus nerve (CSN), restores insulin sensitivity and glucose tolerance in high-energy-fed animal models of insulin resistance and glucose intolerance via a re-establishment of sympathetic nerve activity [4, 5]. Additionally, we described that this re-establishment of metabolic variables was related to the recovery of insulin signalling in insulin-sensitive tissues, as well as the improvement of glucose uptake by the liver and visceral adipose tissue [5]. Herein, we tested the proof-of-principle that inhibition of CSN activity is a sustainable therapeutic strategy for early type 2 diabetes by bilaterally resecting the CSN in rats previously exposed to a high-fat/high-sucrose (HFHSu) diet for 14 weeks. However, knowing in advance that the surgical resection of the CSN is prone to cause side effects related to loss of the peripheral hypoxic response and decreased sensitivity to CO_2_ [6, 7], impaired response to exercise [8–10] and fluctuations in blood pressure [11], we sought to determine whether a reversible approach could induce long-term glycaemic control in rats with diet-induced early diabetes without significant side effects.

A new type therapeutic approach that allows a precise detection and modulation of electrical signalling patterns in the peripheral nervous system, known as bioelectronic medicine, is emerging [12–14]. Acknowledging that the therapeutic approaches currently available for metabolic diseases do not provide long-term control of the disease, combined with significant side effects, a bioelectronic medicine approach could bring significant improvement in the standard of care for type 2 diabetes by targeting nodal metabolic pathways and avoiding systemic effects. Additionally, bioelectronic medicines might have high acceptance among patients given that they require only minimally invasive procedures, while providing high adherence and negligible interference with daily activities [12–15].

In the present work, we tested the use of bilateral kilohertz frequency alternating current (KHFAC) modulation to inhibit CSN activity, since this method has been shown to produce an effective and reversible block of nerve conduction in somatic nerves [16–18]. It has been proposed that the effect of KHFAC is due to direct inhibition of the action potential conduction and that this is achieved within the frequency range 1–100 kHz [16], with the highest frequencies evaluated in vivo being 50 and 70 kHz [19, 20].

## Methods

### Ethical statement

All animal studies were carried out in accordance with the European Union Directive for Protection of Vertebrates Used for Experimental and Other Scientific Ends (2010/63/EU) and the GlaxoSmithKline (GSK) Policy on the Care, Welfare and Treatment of Animals. All surgery was performed following the LASA guiding principles for preparing and undertaking aseptic surgery [21].

### Surgical procedures

Terminal experiments were performed at GlaxoSmithKline (Stevenage, UK) in male Crl:CD(SD) rats (230–280 g, aged 14–16 weeks) obtained from Charles River (Margate, Kent, UK). Recovery experiments were performed at NOVA Medical School Lisbon (Lisbon, Portugal) in male Crl:WI (Wistar Han) rats (200–300 g, aged 8–9 weeks) obtained from the NOVA Medical School animal house. In both animal houses, the rats were kept under temperature and humidity control (21 ± 2°C, 55 ± 10% humidity) with a 12 h light/dark cycle. For recovery experiments animals were blinded divided into two groups: group 1 received a standard chow diet and group 2 were fed an HFHSu diet to induce type 2 diabetes (60% lipid-rich diet plus 35% sucrose in drinking water) over 14–15 weeks. Body weight was periodically recorded, and diet and water consumption were monitored daily. In subsequent experiments, experimenters were blinded to group assignment and outcome assessment. Inclusion criteria for study animals was constant of the insulin tolerance test (K_ITT_) at 14 weeks of diet of 2.8% glucose/min for HFHSu animals and 4.0% glucose/min for control animals. See the electronic supplementary material (ESM) Methods for further details.

#### CSN resection

After insulin sensitivity and glucose tolerance evaluation using the ITT and OGTT, respectively, animals were subjected to bilateral CSN transection under ketamine (30 mg/kg, Nimatek, Dechra, Lostock Gralam, UK) and xylazine (4 mg/kg, Rompun, Bayer, Leverkusen, Germany) anaesthesia and buprenorphine (10 μg/kg, Bupaq, Richter pharma, Wels, Austria) analgesia as previously described [4, 5]. The control groups were subjected to a sham surgical procedure (*n* = 8–10 each group) (ESM Fig. 1).

#### CSN cuff electrode implantation

To evaluate the impact of continuous KHFAC modulation of the CSN, animals from group 1 (non-disease model) and group 2 (diabetes-disease model) were implanted with CSN cuff electrodes. Rats were anaesthetised with urethane (i.p. 1.5 g/kg, Sigma, Gillingham, UK) for acute experiments or medetomidine/ketamine for recovery experiments and bipolar sling cuff electrodes (90% platinum and 10% iridium, 100 μm inner diameter × 1 mm length, electrode surface area 0.4 × 0.5 mm^2^, 0.45 mm interelectrode centre-to-centre distance, CorTec, Freiburg, Germany) were connected to the CSN bilaterally (ESM Fig. 2). Fibrin glue (Tisseel, Baxter Healthcare, Compton, Newbury, UK) was used to secure the cuff to the CSN and to prevent current spread from the ends of the cuff. Wires from the cuff electrodes were tunnelled subcutaneously dorsally on the neck and exteriorised on the head via a skull-mounted percutaneous connector (MS363, Plastics One, Roanoke, VA, USA) and encapsulated with epoxy to form a head cap (ESM Fig. 2). Immediately after implantation, correct electrode placement was confirmed by an increase in respiratory rate during stimulation at 5 Hz and 300 μA for ~2 s. Anaesthesia was reversed with atipamezole (0.25 mg/kg in 2 ml, i.p., Antisedan, Esteve, Finland). Rats were treated postoperatively with analgesic buprenorphine (10 μg/kg, s.c.) and for 2–3 days with anti-inflammatory carprofen (5 mg/kg, s.c, Rimadyl, Pfizer, Zaventem, Belgium). The animals were allowed to recover for 10 days prior to acclimatisation to tethering and initiating the KHFAC modulation.

#### EMG and ECG recording

Intercostal platinum wires for electromyography (EMG) and ECG electrodes were placed subcutaneously across the diaphragm. EMG and ECG data were differentially recorded using Plexon Multichannel Acquisition Processor Data Acquisition System (Plexon, Dallas, TX, USA) and analysed in MatLab.

### KHFAC modulation of the CSN

KHFAC modulation was applied to the cuff electrodes bilaterally as rectangular pulses: at a current of 1 mA for the frequencies 20 kHz, 30 kHz, 40 kHz or at a current of 2 mA for 50 kHz. KHFAC was applied using a commercial current source (Keithley 6221, Tektronix, Bracknell, UK) connected to the percutaneous connector via a tether (305-305, Plastics One). In the percutaneous connector, wires from two cuffs were attached in parallel, so the current was split between the cuffs according to their impedance. To ensure near-equal current split, the cuff electrode impedances were measured in saline (154 mmol/l NaCl) prior to implantation and the cuffs were matched for each animal based on <10% difference in their impedance values. The current values are reported as peak-to-peak for each cuff, assuming equal 50/50% split from the current source output. The current source was only used in the range 0–4.2 mA (peak-to-peak), as higher ranges provided noticeable current attenuation at frequencies about 10 kHz due to poor calibration of the Keithley 6221 device at these frequencies. No capacitors or inductors were used to compensate for possible direct current (DC) offset, so the confounding effect of DC contribution in this study cannot be ruled out. At the same time, omission of output capacitors/inductors avoided distortion of the rectangular pulse shape.

Baseline respiratory frequency and the response to hypoxia (10% O_2_ balanced N_2_) were recorded to determine the effectiveness of KHFAC modulation of the CSN. Respiratory and cardiac variables were measured by intercostal EMG and ECG surface electrodes, respectively.

The effect of continuous KHFAC modulation on insulin sensitivity and glucose tolerance was tested in animals from group 2 (diabetes-disease model). After CSN electrodes implantation, animals from group 2 were randomly divided into two groups. Half of the animals were submitted to continuous KHFAC modulation of the CSN for 9 weeks. KHFAC modulation was not applied to animals in the sham group. To evaluate the reversibility of CSN activity after 9 weeks of KHFAC modulation, the animals were monitored for 5 weeks after cessation of KHFAC modulation for insulin sensitivity, glucose tolerance and ventilatory variables (ESM Fig. 1).

### Experimental design for animal tests

At baseline and before submitting the animals to surgical procedures, animals were evaluated periodically (every 2 or 3 weeks) for fasting glucose, insulin sensitivity and glucose tolerance [5]. After the surgical procedure the animals in group 2 were kept on the HFHSu diet to continue exposure to disease-promoting factors. Fasting glucose, insulin sensitivity, glucose tolerance and weight were evaluated at several time points. Blood was collected from the tail vein at the end of the OGTT to quantify serum mediators. At the terminal experiment, animals were anaesthetised with pentobarbitone (60 mg/kg, i.p.) and mean arterial pressure (MAP) was measured [22]. Blood was then collected by heart puncture for quantification of soluble biomarkers [4, 22]. Fat depots were collected after an abdominal laparotomy and weighted.

#### ITT

Insulin sensitivity was evaluated through an ITT [23] in conscious animals as previously described [4, 5] (see ESM Methods: insulin tolerance test for further details).

#### OGTT

Glucose tolerance was evaluated through an OGTT. The animals were fasted overnight and a bolus of glucose (2 g/kg, Sigma, Madrid, Spain) was administered by oral gavage. Blood samples were collected by modified tail snip at 0, 15, 30, 60, 120 and 180 min intervals (see ESM Methods: oral glucose tolerance test for details).

#### Whole-body plethysmography recordings of ventilation

Ventilation was measured in conscious, freely moving rats by whole-body plethysmography. Tidal volume (VT; ml), respiratory frequency (breaths/min [bpm]) and minute ventilation (VE; ml min^−1^ kg^−1^) were monitored. Protocol consisted of submitting the animals to 20 min acclimatisation followed by 10 min normoxia (20%O_2_ balanced N_2_) followed by 10 min hypoxia (10% O_2_ balanced N_2_), followed by 10 min normoxia, followed by 10 min hypercapnia (20% O_2_ + 5% CO_2_ balanced N_2_) and then 10 min normoxia (see ESM Methods: whole-body plethysmography recordings of ventilation for full details).

#### Quantification of biomarkers: plasma insulin, C-peptide, glucagon, corticosterone, nitric oxide and lipid profile

Insulin and C-peptide concentrations were determined with commercial ELISA kits as previously described [4, 5, 22]. Corticosterone was obtained with DetectX corticosterone immunoassay kit (Arbor Assays, Madrid, Spain). NO/NO_3_^−^ levels were determined in all animals as previously described [4, 22]. The lipid profile was assessed using a RANDOX kit (Irlandox, Porto, Portugal) [5] (see ESM Methods: quantification of biomarkers: plasma insulin, C-peptide, glucagon, corticosterone, nitric oxide and lipid profile for details).

#### Measurement of electrode impedance

Impedance was measured at days 0 and 1 post-implantation and prior to the animal being killed (see ESM Methods: measurement of electrode impedance for details).

#### Histology

Carotid artery bifurcations were collected and processed for microcomputed tomography (microCT) and histology analysis using H&E staining or toluidine blue staining (see ESM Methods: histology for further details).

### Statistical analysis

Statistical analyses were performed using GraphPad Prism software, version 6 (GraphPad Software, La Jolla, CA, USA) and by MatLab Statistics and Machine Learning Toolbox, version 8.5 (Natick, MA, USA). The significance of the differences between the mean values was calculated by one- and two-way ANOVA with Dunnett’s and Bonferroni multicomparison test, respectively. Differences were considered significant at *p* < 0.05.

## Results

### Effect of chronic bilateral CSN resection in an animal model of diet-induced type 2 diabetes

We have previously demonstrated that chronic bilateral resection of CSN reverses insulin resistance and glucose intolerance in rats with metabolic syndrome [4, 5]. However, from a clinical perspective, the modulation of CSN would have a higher impact if it showed beneficial effects in an animal model of type 2 diabetes. Herein we have used a model of type 2 diabetes obtained by submitting Wistar rats to a HFHSu diet for 14 weeks. Bilateral chronic CSN resection restored insulin sensitivity to baseline values (see ESM Fig. 3a–c), an effect that was preserved over 11 weeks post-resection. Furthermore, CSN resection restored fasting glucose (ESM Table 1) and improved glucose tolerance (ESM Fig. 3d–f, ESM Table 2), and fasting plasma insulin and C-peptide (ESM Table 1). CSN resection also normalised mean blood pressure and nitric oxide levels (ESM Fig. 3h), as well as LDL-cholesterol and triacylglycerols (ESM Table 3). The surgical intervention had no effect on metabolic and haemodynamic variables in the group fed a standard diet (ESM Fig. 3a–h). All together, these results demonstrate that bilateral CSN resection improves insulin sensitivity and glucose metabolism in a model of type 2 diabetes.

However, the surgical transection of these peripheral nerves is unlikely to be well-accepted by patients or clinicians. Therefore, we envisaged and applied a novel modality of ‘on demand’ and reversible inhibition of the nerve through KHFAC.

### Establishing KHFAC parameters for CSN inhibition

**Fig. 1:**
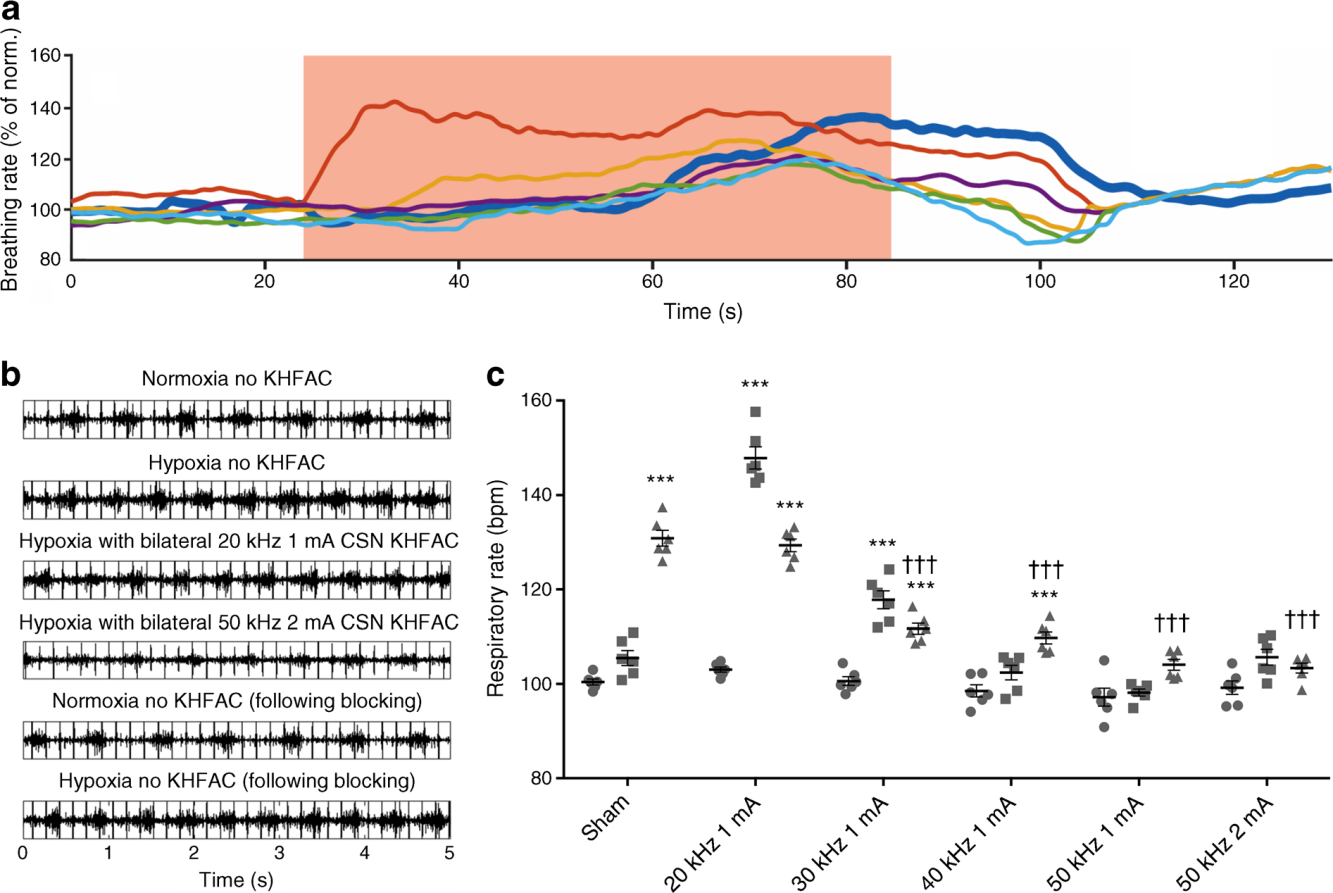
Effect of KHFAC modulation of the CSN on cardiorespiratory responses to hypoxia. (**a**) Cardiorespiratory responses to hypoxia over time with and without KHFAC modulation (period of hypoxia shown by red shaded area). Dark blue, no KHFAC modulation; orange, 20 kHz/1 mA; yellow, 30 kHz/1 mA; purple, 40 kHz/1 mA; green, 50 kHz/1 mA; light blue, 50 kHz/2 mA. Norm., normoxia. (**b**) Raw data traces of EMG and ECG in normoxic and hypoxic conditions with and without KHFAC modulation. (**c**) Quantification of respiratory rate change in response to hypoxia with and without KHFAC modulation. Data points represent individual animals and bars represent overall mean respiratory frequency. Circles, baseline; squares, first 30 s of hypoxia response; triangles, last 30 s of hypoxia response. Data represent means ± SEM. One- and two-way ANOVA with Bonferroni multicomparison test: ***p < 0.001 vs baseline within the stimulation group; ^†††^*p* < 0.001 vs sham

We established the KHFAC modulation parameters required to inhibit CSN activity by testing the effect of KHFAC on the classical physiological stimulus of the carotid body: hypoxia [24]. A 1 min exposure to hypoxic air (10% O_2_ + 90% N_2_) resulted in a reproducible and significant (*p* = 3.04 × ^−10^) increase in respiratory rate from baseline to about 30–60 s after induction in the sham group (100.4 ± 1.55 bpm vs 130.9 ± 4.08 bpm) (Fig. 1a, b). Notably, no significant difference was seen over the first 30 s of hypoxia, probably due to the time required for blood saturation (Fig. 1). KHFAC, applied for 1 min (increasing in frequency from 30–50 kHz), significantly reduced the respiratory response to hypoxia compared with the sham group (30 kHz, 111.7 ± 2.87; 40 kHz, 109.8 ± 3.13; 50 kHz, 104.1 ± 2.86; 50 kHz at 2 mA, 103.4 ± 2.54) (Fig. 1a–c). Figure 1c shows a dose–response relationship between the frequency of KHFAC and efficiency at preventing the respiratory effects of hypoxia. Frequencies of 30 kHz and 40 kHz did not provide a 100% suppression of the respiratory rate, as evidenced by a remaining significant difference in ventilatory response in the final 30 s compared with baseline. However, at 50 kHz, the respiratory rate during hypoxia was not significantly different from baseline, indicating an almost complete suppression.

At 20 kHz, no inhibition of respiration frequency was observed compared with sham levels. Furthermore, at 20 kHz an exacerbation of the hypoxic respiratory response occurred over the first 30 s (43.5 ± 6.29%) significantly higher than in sham stimulations (*p* < 0.05) (Fig. 1c), suggesting neural activation rather than inhibition. A smaller exacerbation in response was seen at 30 kHz. The KHFAC applied at 50 kHz and 2 mA produced the most robust inhibition of cardiorespiratory responses to hypoxia without any onset cardiac or respiratory responses (Fig. 1c); therefore, 50 kHz was used for long-term KHFAC modulation to provide superior safety and efficacy.

After each KHFAC application, the reversibility of its effect on the CSN was confirmed by the recovery of respiratory responses to hypoxia (Fig. 1b). KHFAC modulation had no effect on respiratory response during normoxic air breathing (data not shown).

These results demonstrate that KHFAC modulation applied at specific frequencies and amplitudes, specifically 40–50 kHz at 1–2 mA, can suppress the hypoxia response without causing an abnormal physiological onset response, and that the observed effect is fully reversible.

### Effect of chronic KHFAC modulation of the CSN on animal behaviour and ventilation

Previously, there have only been a few studies on the effects of chronic KHFAC modulation [18–20], none of which reported the effect on the CSN. To evaluate the impact of KHFAC modulation on the CSN, 50 kHz square pulses at 2 mA (peak-to-peak) were applied continuously for 1 week in healthy animals fed a standard diet. No behavioural alterations were observed and the animals responded well to headcaps and tethers. Following cessation of KHFAC modulation, animals were exposed to a hypoxic stimulus (10% O_2_ balanced N_2_) and the respiratory response was evaluated at two time points: 20 min and 1 week post-KHFAC. At 20 min post-KHFAC, animals presented a decrease in basal ventilation and did not respond to the hypoxic challenge, demonstrating that a functional neuromodulation was maintained (ESM Fig. 4). At 1 week post-KHFAC, the basal ventilation and the response to hypoxia returned to baseline levels (increase in respiratory rate of ~20%) (ESM Fig. 4). The decrease in basal ventilation and abolishment of hypoxic ventilatory responses demonstrate that suppression of the carotid body response was produced in conscious animals via KHFAC modulation of the CSN. Reversibility of the suppression was also demonstrated by the recovery of hypoxia sensitivity, suggesting that long-term application of KHFAC did not cause permanent pathological changes in the nerve conduction properties.

### Effect of chronic KHFAC modulation of the CSN on insulin sensitivity and glucose homeostasis and its reversibility in an animal model of diet-induced type 2 diabetes

**Fig. 2:**
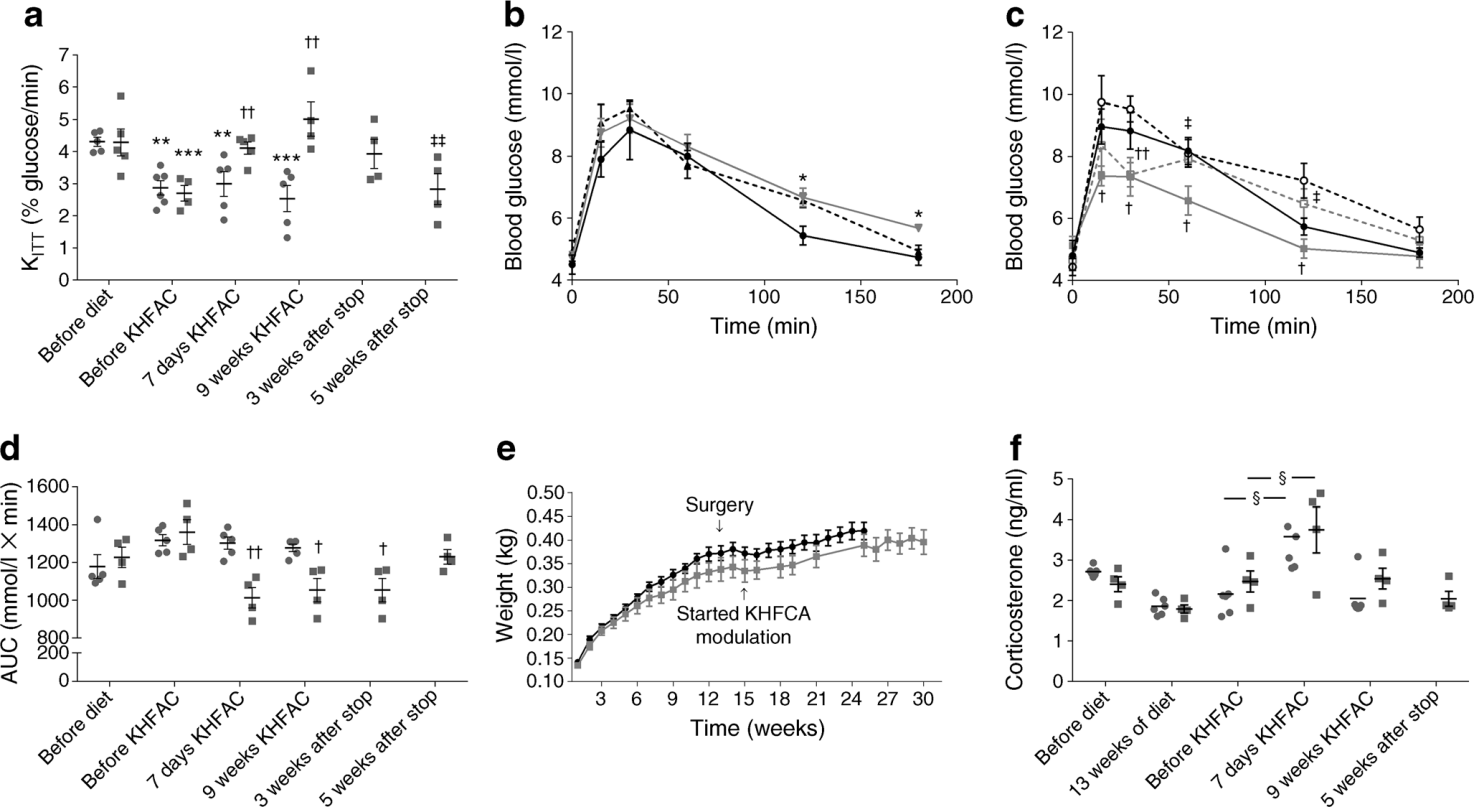
Effect of KHFAC modulation of the CSN on cardiometabolic variables and stress responses in HFHSu-induced type 2 diabetes and its reversibility. (**a**) Effect of KHFAC on insulin sensitivity assessed by an ITT and expressed as the constant rate for glucose disappearance (K_ITT_) in HFHSu-fed animals. (**b**) Glucose excursion curves in HFHSu sham animals before diet (black circles), after 15 weeks of diet (grey triangles) and at 24 weeks of diet (black triangles). (**c**) Glucose excursion curves in HFHSu KHFAC animals before diet (black circles), prior to the initiation of KHFAC (white circles), during 9 weeks of KHFAC (grey squares), and 5 weeks post-KHFAC (white squares). (**d**) Effect of KHFAC modulation on the AUC obtained through the analysis of glucose excursion curves. (**e**) Effect of KHFAC modulation on weight gain curves in HFHSu sham (black line) and HFHSu KHFAC (grey line) animals. (**f**) Effect of KHFAC modulation of the CSN on corticosterone levels in HFHSu-fed animals. Sling cuffs were implanted at week 13 of the HFHSu diet; 2 weeks post-surgery KHFAC stimulation was started. Sham animals represent HFHSu-fed animals with electrical cuff implants but no KHFAC stimulation. (**a**, **d**, **f**) Circles, HFHSu sham; squares, HFHSu KHFAC. Data are means ± SEM of 4–5 animals. One- and two-way ANOVA with Dunnett’s and Bonferroni multicomparison tests: *p < 0.05, **p < 0.01, ***p < 0.001 vs chow-fed controls; ^†^*p* < 0.05, ^††^*p* < 0.01 vs sham; ^‡^*p* < 0.05, ^‡‡^*p* < 0.01 vs 9 weeks of KHFAC; § before KHFAC vs 7 days of KHFAC

Effect of KHFAC modulation of the CSN on insulin sensitivity and glucose homeostasis was evaluated in HFHSu-fed animals (Fig. 2a–f, Table 1). As expected, HFHSu-fed animals were insulin-resistant (data not shown) and exhibited increased fasting insulin and C-peptide levels (Table 1). Electrode implantation on the CSN had minimal impact upon K_ITT_ (Fig. 2a; K_ITT_ HFHSu sham, 2.87 ± 0.22% glucose/min; HFHSu KHFAC, 2.70 ± 0.24% glucose/min) and on fasting insulin and C-peptide levels (Table 1) measured 2 weeks post-implantation. Furthermore, glucose tolerance post-surgery, measured by an OGTT, confirmed that the glycaemic response to a stimulus was not impacted by implantation surgery (Fig. 2b, c).

**Table 1:** Effects of KHFAC modulation of the CSN on fasting blood glucose, insulin and C-peptide in HFHSu-fed animals

	Group of animals	Before diet	13 weeks of diet	Before KHFAC	9 weeks of KHFAC	5 weeks after KHFAC
Blood glucose (mmol/l)	HFHSu sham	4.14 ± 0.31	5.06 ± 0.22*	4.83 ± 0.24	6.15 ± 0.26^***,†^	
	HFHSu KHFAC	4.45 ± 0.22	5.24 ± 0.32	4.96 ± 0.47	5.54 ± 0.32	4.83 ± 0.23
Insulin (pmol/l)	HFHSu sham	99.69 ± 4.65	388.34 ± 25.83*	372.95 ± 31.63*	488.83 ± 107.45**	
	HFHSu KHFAC	103.41 ± 6.47	426.38 ± 13.45*	445 ± 61.21**	410.48 ± 72.71*	425.81 ± 90.26*
C-peptide (nmol/l)	HFHSu sham	0.44 ± 0.44	1.22 ± 0.27*	1.37 ± 0.133*	2.45 ± 0.34**	
	HFHSu KHFAC	0.50 ± 0.05	1.12 ± 0.14*	1.22 ± 0.15*	2.33 ± 0.34**	2.675 ± 0.18**

Data are means ± SEM of 4–5 animals

One- and two-way ANOVA with Dunnett’s and Bonferroni multicomparison test: *p < 0.05, **p < 0.01, ***p < 0.001 vs chow-fed controls; ^†^*p* < 0.05 vs HFHSu KHFAC

The electrical impedance spectroscopy at 1 day post-implantation confirmed the stability of the cuff–nerve interface, with the average impedance of 8.8 ± 1.5 kΩ at 1 kHz (ESM Fig. 5).

**Fig. 3:**
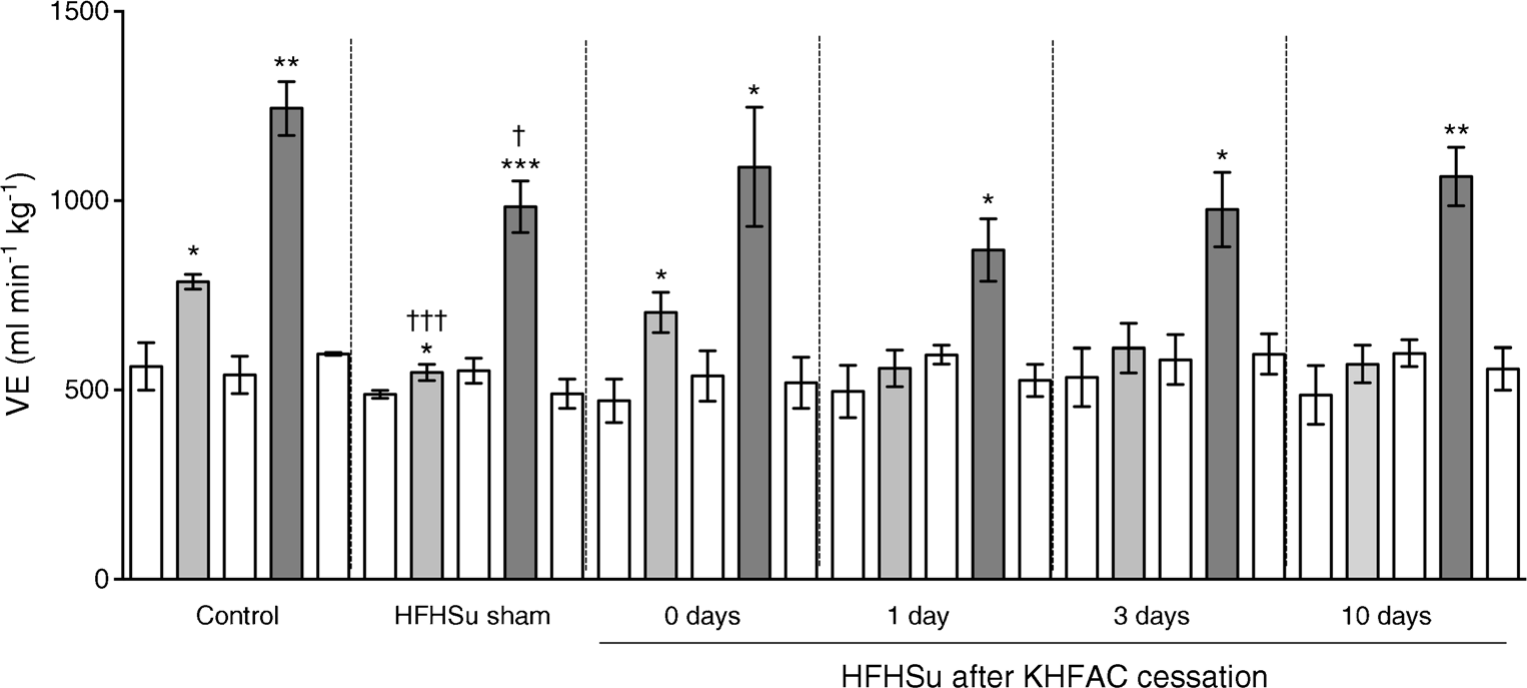
Impact of KHFAC modulation of the CSN on ventilatory responses to hypoxia and hypercapnia in age-matched chow-fed control, HFHSu sham and HFHSu KHFAC animals. Ventilatory recordings were performed immediately post-KHFAC and at 1 day, 3 days and 10 days after cessation of KHFAC. Ventilatory recordings were performed in freely moving animals and the protocol consisted of 10 min normoxia (20% O_2;_ white bars), followed by 10 min hypoxia (10% O_2_; light grey bars), followed by 10 min normoxia, followed by 10 min hypercapnia (20% O_2_ + 5% CO_2_; dark grey bars) and finally by 10 min normoxia. Sham animals represent HFHSu animals with electrical cuffs implanted but not submitted to KHFAC. Data are means ± SEM of 4–5 animals. One- and two-way ANOVA with Dunnett’s and Bonferroni multicomparison test: *p < 0.05, **p < 0.01, ***p < 0.001 vs normoxia applied immediately before hypoxic or hypercapnic stimuli; ^†^*p* < 0.05, ^†††^*p* < 0.001 vs age-matched control animals

HFHSu-fed animals were then randomised and KHFAC modulation was applied in half of the animals. After 1 week of KHFAC, a significant increase in insulin sensitivity (Fig. 2a) and a decrease in glucose intolerance was observed (Fig. 2b, c), with no effects on insulin or C-peptide levels (Table 1). These effects on glucose metabolism were maintained over 9 weeks of KHFAC modulation (K_ITT_ HFHSu sham, 2.56 ± 0.41%glucose/min; K_ITT_ HFHSu KHFAC, 5.01 ± 0.52%glucose/min; AUC glucose excursion curve HFHSu sham, 1278 ± 20.36 mmol/l × min; AUC glucose excursion curve HFHSu KHFAC, 1054.15 ± 62.64 mmol/l × min) and were subsequently reversed over the 5 weeks following cessation of KHFAC, evidenced by the re-emergence of insulin resistance and glucose intolerance (Fig. 2a–f, Table 1). Full reversibility of KHFAC-induced suppression of respiratory responses to hypoxia was observed at 10 days after the cessation of KHFAC modulation (Fig. 3, Table 2).

**Table 2:** Effects of KHFAC modulation of the CSN on basal ventilation (frequency, VT and VE) in chow-fed controls and in HFHSu-fed animals with or without KHFAC modulation

	Control	HFHSu sham	HFHSu KHFAC^a^
Frequency (bpm)	83.38 ± 7.65	84.68 ± 6.44	81.20 ± 6.32
VT (ml/kg)	6.72 ± 0.28	5.67 ± 0.33	6.06 ± 0.33
VE (ml min^−1^ kg^−1^)	562.0 ± 62.3	488.5 ± 10.6	471.5 ± 57.7

Data are means ± SEM of 4–5 animals

^a^Basal ventilatory recordings were performed immediately post-KHFAC

Corticosterone levels were evaluated periodically to assess the stress response to KHFAC modulation. As seen in Fig. 2f, corticosterone levels were increased 1 week after initiation of KHFAC modulation. This effect, however, cannot be directly attributed to KHFAC, as the non-stimulated sham group also presented an increase in hormonal levels. Furthermore, at 9 weeks after KHFAC modulation, corticosterone values returned to the baseline level and remained low after cessation of KHFAC. No behavioural alterations were observed.

### Effect of chronic KHFAC modulation on the axonal and myelin integrity of the CSN

**Fig. 4:**
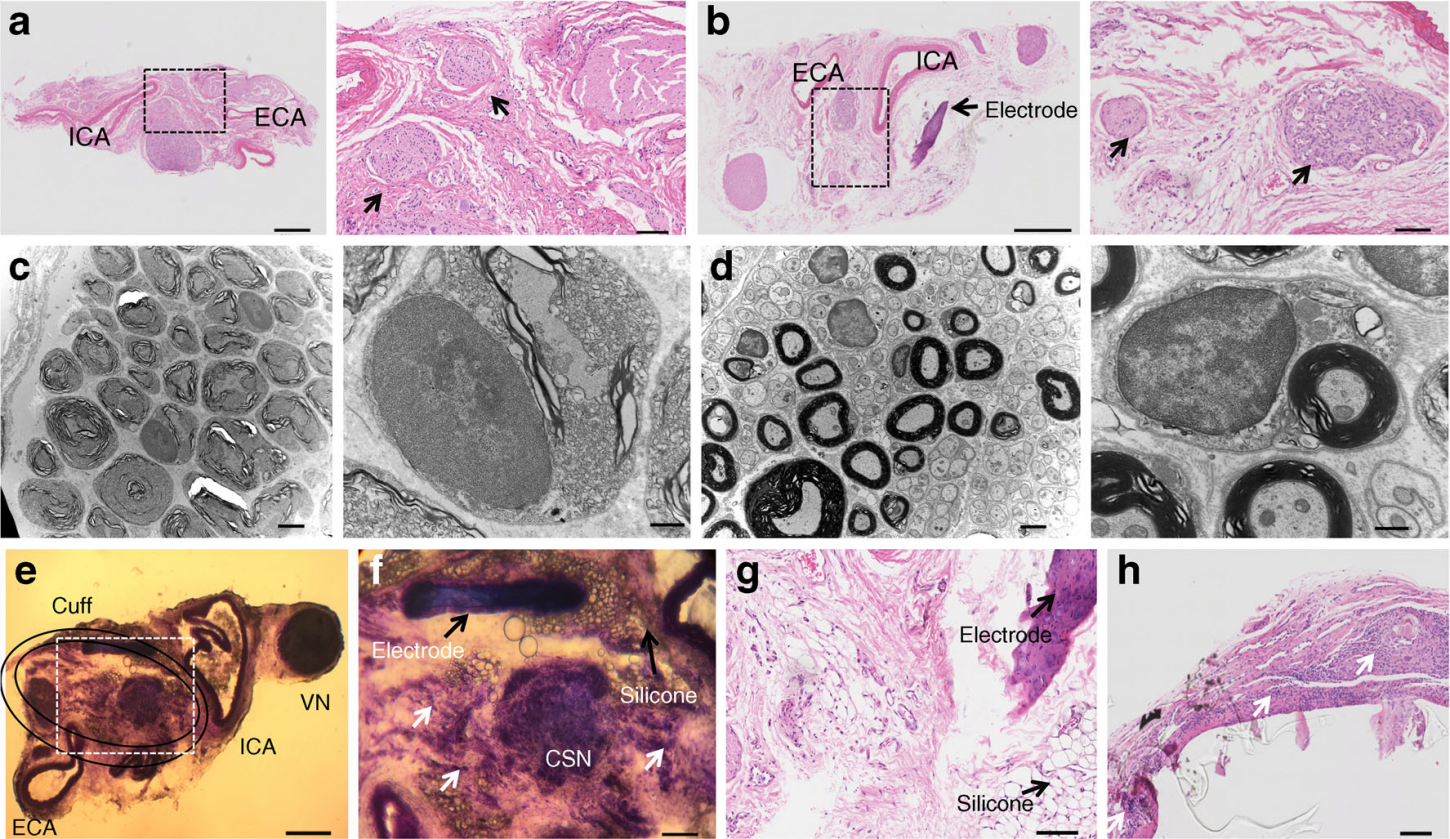
Impact of KHFAC modulation of the CSN on CSN histology. Low and high magnification pictures of 4 μm thick sections stained with haematoxylin–eosin of the left and right carotid bifurcation area isolated from rats in the (**a**) HFHSu sham group and (**b**) HFHSu KHFAC group (scale bars, 500 μm and 100 μm for low and high magnification, respectively); black arrows indicate putative CSN sections. Electron microscopy images of the CSN at low and high magnification of the left and right CSN isolated from rats in the (**c**) HFHSu sham group and (**d**) HFHSu KHFAC group (scale bars, 2 μm and 500 nm for low and high magnification, respectively). (**e**) Low magnification image of a 30 μm thick section of the carotid bifurcation area, stained with toluidine blue, isolated from an HFHSu animal implanted with an electrical cuff (scale bar, 500 μm). (**f**–**h**) High magnification images of the carotid bifurcation area (scale bars, (**f**) 200 μm; (**g**, **h**), 100 μm); black arrows roughly indicate the position of the implanted cuff as well as fragments of electrodes and silicone; white arrows indicate areas of fibrosis characterised by high density of cells. ECA, external carotid artery; ICA, internal carotid artery; VN, vagus nerve

Correct placement of and tissue response to the sling cuffs were evaluated post-mortem by light and electron microscopy, histology and by contrast microCT imaging. The microCT imaging was used in HFHSu sham and HFHSu KHFAC animals to visualise placement of the electrical cuffs on the CSN in the dissected tissue block with common carotid artery bifurcation (ESM Fig. 6). Next, a qualitative histology analysis was performed to evaluate possible damage to the CSN by a foreign body response to the implanted cuffs and long-term application of KHFAC. Transverse haematoxylin/eosin staining of sections from sham-stimulated animals evidenced no discernible signs of the nerve damage (Fig. 4a, b). In Fig. 4b intact nerve tissue can be seen in intimate contact with the sling cuff around the CSN. Evaluation of the CSN adjacent to the cuff by electron microscopy indicated a similarly minor amount of myelin damage in both the sham and stimulated animals (Fig. 4c, d). In Fig. 4e–h, frozen and paraffin-embedded sections stained with toluidine blue and haematoxylin/eosin, respectively, demonstrate long-term infiltration of immune cells (e.g. macrophages and neutrophils), as well as chronic adipose tissue deposition that occurs during the foreign body reaction process to implanted material (see white and black arrowheads in Fig. 4f–h).

## Discussion

We describe that electrical modulation of the CSN restores metabolic homeostasis in an animal model of early type 2 diabetes. Neuromodulation of the CSN was achieved by application of bilateral KHFAC through surgically implanted cuff electrodes. The beneficial effects of KHFAC on glucose tolerance and insulin sensitivity were reversed after discontinuation of the electrical stimulus. Together, these findings support a potential role for bioelectronic medicines in the treatment of type 2 diabetes.

In this study, we provide further evidence that CSN overactivity is linked to the development of diet-induced insulin resistance and glucose intolerance [4, 5]. The carotid body chemoreceptors have been previously associated with the aetiology of cardiometabolic diseases both in animal models [25–27] and in humans [28–30].

Approaches that specifically target the carotid body may represent a putative therapeutic strategy to pharmacological-treatment-resistant metabolic diseases. However, surgical procedures that are currently offered present several drawbacks. Unilateral ablation of a single carotid body has been shown to reduce arterial pressure in individuals with hypertension; however, efficacy is diminished 12 months after ablation, suggesting a compensation of the remaining carotid body and the need for bilateral ablation to achieve a sustained therapeutic effect [30]. Additionally, bilateral surgical removal of the carotid bodies or the CSN is prone to side effects related to both the chemosensory and baroreceptor functions of the carotid sinus. Furthermore, even bilateral ablation may be non-sustainable in the long term, since the CSN nerve fibres regenerate with time. Herein, we show that bilateral KHFAC modulation of the CSN may be an alternative approach since it mimics the beneficial metabolic effects of the bilateral surgical resection of the CSN combined with the advantages of being tuneable and fully reversible.

KHFAC modulation at 10–70 kHz has been suggested as an effective method to suppress nerve conduction, since action potentials are arrested as they pass the depolarising charge field of the electrode [18, 20]. In this study, we demonstrated that acute application of KHFAC induces nearly complete inhibition of the relevant physiological response: decreased ventilation and hypoxic response. The effect of KHFAC modulation lasted for 20 min and caused no permanent nerve damage, as indicated by a full recovery of ventilation and hypoxic responses to pre-KHFAC values at 1 week after cessation of KHFAC modulation. The ‘onset response’, a transitory end-organ activation that commonly occurs at 10 kHz (and below) in motor nerves [31, 32], was evident as an enhanced cardiorespiratory response at 20 kHz modulation of the CSN, less evident at 30 kHz and not observed at 50 kHz. Also, the stress response to 9 week KHFAC modulation of the CSN at 50 kHz with a 2 mA current was minimal, as assessed by corticosterone plasma levels.

Effective nerve modulation with KHFAC requires an electrode design that guarantees a uniform delivery of current to the nerve. Herein, we used sling cuff electrodes consisting of an insulating outer layer and nearly circumferential metal electrode contacts inside the cuff, as similar cuffs have been shown to be effective in KHFAC modulation of other nerves [16, 33]. The long-term stability of the cuff placement was confirmed by qualitative histology analysis and by evaluating electrode impedance. Reversibility of KHFAC was also demonstrated, since the CSN recovers its ability to conduct action potentials 1 week after cessation of chronic KHFAC pulsing.

A lower KHFAC frequency of 5 kHz has been evaluated in human clinical trials. Intermittent KHFAC modulation (5 min on and 5 min off, amplitudes ranging from 1–6 mA) of the intra-abdominal vagus nerve, using an implanted spiral cuff has been recently trialled in nearly 200 individuals as a therapy for appetite suppression and obesity control [15, 34]. Subjects were followed for 1 year and their weight loss was found to be linearly correlated with the duration of KHFAC modulation. There were no significant adverse events related to the KHFAC therapy. KHFAC modulation has also been used for temporary (10 min) pain relief with a cuff placed on the sciatic and tibial nerves [35]. Therefore, the use of KHFAC modulation could provide an effective and safe option for treatment of various chronic disorders.

In conclusion, the present study shows that KHFAC modulation of the CSN improves metabolic control in a rodent model of early type 2 diabetes, an effect that is long-lasting and that persists despite the continued influence of disease-promoting factors, such as a high-energy diet. According to our results, KHFAC modulation of the CSN has significant potential as a means of controlling CSN activity for the purpose of treating metabolic diseases.

## Electronic supplementary material

ESM (PDF 1202 kb)

## Data Availability

All data generated or analysed during this study are included in this published article (and its supplementary information files).

## Abbreviations

bpm: Breaths per min
CSN: Carotid sinus nerve
DC: Direct current
EMG: Electromyography
HFHSu: High-fat/high-sucrose
KHFAC: Kilohertz frequency alternating current
K_ITT_: Constant of the insulin tolerance test
MAP: Mean arterial pressure
microCT: Microcomputed tomography
VE: Minute ventilation
VT: Tidal volume
