# Supplementary material for: Bioelectronic modulation of carotid sinus nerve activity in the rat: a potential therapeutic approach for type 2 diabetes
Source: Diabetologia. 2018 Jan 14;61(3):700–10. doi: 10.1007/s00125-017-4533-7 (PMC6448966; doi:10.1007/s00125-017-4533-7)
Supplement: Supplementary file 1 — (PDF 1202 kb) [file 125_2017_4533_MOESM1_ESM.pdf]

Electronic Supplementary Material

ESM Methods

Surgical procedures

Terminal experiments were performed at GlaxoSmithKline, UK in Male Crl:CD(SD) rats (230-280g, aged 14-16 weeks) obtained from Charles River, Kent, UK. Pre-operatively, the animals were fed “ad libitum” Purina Mills International 5CR4 Rodent Diet and animal grade drinking water, (Veolia Water supply, London, UK). Animals were maintained under temperature and humidity control of (21±2°C; 55 ±10% humidity) with a 12h light-12h dark cycle. Suitable environmental enrichment was provided.

Recovery experiments were experiments performed at NOVA Medical School Lisbon, Portugal. Male Crl:WI (Wistar Han) rats (200-300g, aged 8-9 weeks) were obtained from the animal house of the NOVA Medical School. Animals were group housed prior to study and kept under temperature and humidity control (21±2°C; 55±10% humidity) with a 12h light-12h dark cycle. Environmental enrichment was provided in the form of cardboard tubes and wood chew blocks. For recovery studies animals were randomly divided into two groups: Group 1 that was submitted to a standard diet (7.4% fat, 17% protein and 75% carbohydrate (4% sugar) (Dietex International Limited, France) and Group 2 that fed High-Fat High-Sucrose (HFHSu) to induce T2D (60% lipid rich diet: 34% fat+33% carbohydrate+23% protein; Mucedola, Milan, Italy) plus sucrose (35%) in drinking water) during 14-15 weeks. Body weight were periodically recorded and diet and water consumption were monitored daily.

Experimental design for animal tests

Insulin Tolerance Test

Insulin sensitivity was evaluated through an insulin tolerance test (ITT) in conscious animals. The ITT is one of the earliest methods developed to assess insulin sensitivity in vivo and provides an estimate of overall insulin sensitivity, correlating well with the ‘gold standard’ hyperinsulinaemic–euglycaemic clamp [23]. The ITT consists in the administration of an intravenous insulin (Humulin® R 100IU/ml, Lilly) bolus of 0.1 U/kg body weight in the tail vein, after an overnight fast (approx. 16 hours), followed by the measurement of the decline in plasma glucose concentration over a 15 minute period. The constant rate for glucose decline (K_{ITT}) was calculated using the formula $0.693/t_{1/2}$ [22,23]. Glucose half-time ($t_{1/2}$) was calculated from the slope of the least square analysis of plasma glucose concentrations during the linear decay phase. Blood samples were collected by modified tail snip technique and glucose levels were measured with a glucometer (Precision Xtra Meter, Abbott Diabetes Care, Portugal) and test strips (Abbott Diabetes Care, Portugal) [4,5].

Oral Glucose Tolerance test

Glucose tolerance was evaluated through an oral glucose tolerance test. For that, the animals were fasted overnight and a bolus of glucose (2g/kg, Sigma, Madrid, Spain) was administered by oral gavage. In animals with electrodes implanted at the CSN, in order to protect the CorTec™ electrode wires running subcutaneously from the headcap dorsally round to the CSN, the animals were orally dosed for the OGTT test using a flexible rubber nelaton dosing catheter, model 7.17.1; 10 French g, 39 cm long (Harvard Apparatus UK, Cambridge,

UK). This allowed a suitable handling and restraint method for dosing procedure, eliminating the need to scruff the animals and prevented damage to the subcutaneous electrodes. Blood samples were collected by modified tail snip at intervals of 0, 15, 30, 60, 120, and 180 minutes. Glucose levels were measured with a glucometer (Precision Xtra Meter, Abbott Diabetes Care, Portugal) and test strips (Abbott Diabetes Care, Portugal).

Whole-body plethysmography recordings of ventilation

Ventilation was measured in conscious freely moving rats by whole-body plethysmography. The system (Emka Technologies, Paris, France) consisted of 5-litre methacrylate chambers continuously fluxed (2 l/min) with gases. Tidal volume (VT; ml) respiratory frequency (f; breaths/min; bpm) and minute ventilation (VE; ml/min/Kg) were monitored. Each rat was placed in the plethysmography chamber and allowed to breathe room air for 20 min to allow adaptation to chamber environment and to acquire a standard resting behavior. Animal acclimatized well during this period and enabled subsequent ventilatory parameters to be recorded according to the protocol used. The protocol consisted in submitting the animals to 20 mins acclimatization followed by 10 min normoxia (20% O_2 balanced N_2) followed by 10 min hypoxia (10% O_2 balanced N_2), followed by 10 min normoxia, followed by 10 min hypercapnia (20% O_2 + 5% CO_2 balanced N_2) and then 10 min normoxia. The pressure change within the chamber reflecting tidal volume (VT) was measured with a high-gain differential pressure transducer. Ideally, the frequency of pressure fluctuations is identical to breathing movements; spurious fluctuations of the pressure due to animal movements were electronically rejected. The amplitude

of the pressure oscillations is proportionally related to VT; a calibration of the system by injections of 0.2 to 0.5 ml air into the chamber allowed a direct estimation of VT. Pressure signals were fed to a computer for visualization and storage for later analysis with EMKA software (Emka Technologies, Paris, France).

Quantification of biomarkers: plasma insulin, C-peptide, glucagon, corticosterone, nitric oxide and lipid profile

Insulin and C-peptide concentrations were determined with an enzyme-linked immunosorbent assay (ELISA) kit (Mercodia Ultrasensitive Rat Insulin ELISA Kit and Mercodia Rat C-peptide ELISA Kit, respectively, Mercodia AB, Uppsala, Sweden). Glucagon and corticosterone determination was obtained with a enzyme immunoassay kit RayBio GLU EIA kit and DetectX corticosterone immunoassay kit, respectively (Arbor Assays, Madrid, Spain). To evaluate endothelial function and inflammation, plasma NO/NO3- levels were determined in all animals [4,22]. For that, proteins from serum samples were precipitated by adding two volumes of ethanol (0ºC). After 30 minutes on ice, samples were centrifuged in a microfuge (Eppendorf, Madrid, Spain) at 12000g for 10 minutes. NO/NO_3^- concentration was determined by using a specific and sensitive NO/ozone chemiluminescence technique (NO-Analyzer 280, Sievers Research Inc., Boulder Colorado). The lipid profile was assessed using a RANDOX kit (RANDOX, Irlandox, Porto, Portugal) to determine total cholesterol and triglycerides by Trinder-based colorimetric end-point assays, and high-density lipoprotein (HDL) and low-density lipoprotein (LDL) by a direct-HDL and direct-LDL clearance method, respectively.

Measurement of electrode impedance

Impedance was measured at 0 and 1 days post-implantation and just prior to animal's sacrifice using a handheld potentiostat (pocketSTAT, Ivium Technologies B.V., Eindhoven, Netherlands) connected to a computer running the electrochemical impedance spectroscopy (EIS) software (IviumSoft, Ivium Technologies). The impedances were measured using a two-pole setup: working and sense electrodes were connected to form the first pole, while the counter and reference electrodes were connected to form the second pole, ground electrode not connected. The EIS was performed at frequencies from 10 to 10,000Hz (ESM Fig. 4).

Histology

For histology analysis, the carotid artery (CA) area and the cuff electrodes were bilaterally dissected and immersion-fixed in PFA 4%. Samples were embedded into paraffin for routine H&E staining (4µm thick coronal sections). Some samples were instead frozen and cut with a cryostat (20-30µm thick) for H&E or toluidine blue staining. Some samples were used for electron microscopy analysis. Fixed samples were post fixed in 1% aqueous osmium tetroxide and processed into Agar 100 resin.1 mm toluidine blue stained survey sections were prepared and examined by light microscopy to locate the areas of interest. Ultra-thin sections (1-µm thick) were stained with uranyl acetate and lead citrate and examined in a Hitachi H7500 transmission electron microscope. The AMT XR41 Digital Camera System v600.202 was used to capture TEM digital images. Representative digital images were taken.

For Micro CT analysis, fixed samples were incubated in 0.3% phosphotungstic acid solution (in 70%EtOH). Contrast images were then acquired with a SkyScan1176 (Bruker microCT, US) at 18μm resolution. Images were then reconstructed using NRecon Software v1.6.10.4.

ESM Tables

ESM Table 1 – Effect of carotid sinus nerve (CSN) bilateral resection on fasting blood glucose and insulin and C-peptide in control (CTL) and early-type 2 diabetes (HFHSu) animals.

Treatments	Models	Baseline	14 weeks of diet	25 weeks of diet
**Blood glucose (mmol/l)**	CTL Sham	5.08 ± 0.13	4.60 ± 0.13	4.81 ± 0.13
	CTL – CSN resection	5.07 ± 0.10	4.86 ± 0.29	4.74 ± 0.30
	HFHSu Sham	4.80 ± 0.15	5.40 ± 0.10***	5.47 ± 0.19***
	HFHSu – CSN resection	4.80 ± 0.19	5.38 ± 0.12**	4.90 ± 0.16†,‡
**Insulin (pmol/l)**	CTL Sham	98.0 ± 19	161.0 ± 19.5*	199.6 ± 23.3*
	CTL – CSN resection	103.5 ± 20.8	190.2 ± 27.2*	191.9 ± 49.2*
	HFHSu Sham	110.9 ± 17.1	421.8 ± 62.5***	595.9 ± 116.4***
	HFHSu – CSN resection	104.6 ± 18.3	370.0 ± 32.1***	381.8 ± 41.7***,‡‡
**C-peptide (nmol/l)**	CTL Sham	0.42 ± 0.08	0.65 ± 0.08**	0.85 ± 0.10**
	CTL – CSN resection	0.43 ± 0.13	0.79 ± 0.12**	0.76 ± 0.16**
	HFHSu Sham	0.44 ± 0.09	1.57 ± 0.27***	2.03 ± 0.39***
	HFHSu – CSN resection	0.43 ± 0.08	1.54 ± 0.19***	1.89 ± 0.30***

Data are means ± SEM of 8/10 animals. One and Two-Way ANOVA with Dunnet's and Bonferroni multicomparison test: *p<0.05, **p<0.01, ***p<0.001 *vs* control; †p<0.05, with *vs* without CSN resection; ‡p<0.05, ‡‡p<0.01 with *vs* without CSN resection at 25 weeks of diet.

ESM Table 2 – Area under the curve (AUC) obtained through the analysis of the glucose excursion curves in control (CTL) and early-type 2 diabetes (HFHSu) animals with or without carotid sinus nerve (CSN) bilateral resection.

Area under curve (mmol/l x min)	Baseline	14 weeks of diet	25 weeks of diet
**CTL Sham**	1254 ± 27	1233 ± 26	1192 ± 25
**CTL – CSN resection**	1276 ± 17	1193 ± 43	1141 ± 58
**HFHSu Sham**	1256 ± 38	1387 ± 28**	1372 ± 31**
**HFHSu – CSN resection**	1252 ± 29	1353 ± 26**	1263 ± 22†

Data are means ± SEM of 8/10 animals. One and Two-Way ANOVA with Dunnet's and Bonferroni multicomparison test: ***p*<0.01, *vs* control; †*p*<0.05, with *vs* without CSN resection.

ESM Table 3 – Effect of carotid sinus nerve (CSN) bilateral resection on total, visceral/perienteric, epididymal and perinephric fat and on the lipid profile (total cholesterol, HDL, LDL and triacylglycerols) in control (CTL) and early-type 2 diabetes (HFHSu) animals.

	CTL Sham	CTL – CSN resection	HFHSu Sham	HFHSu – CSN resection
**Total fat (g/kg)**	66.44 ± 3.93	64.14 ± 5.16	113.9 ± 9.73***	104.2 ± 7.64
**Perienteric fat (g/kg)**	15.58 ± 0.82	14.42 ± 1.37	24.44 ± 2.80**	22.97 ± 1.74
**Perinephric fat (g/kg)**	25.78 ± 1.75	26.03 ± 1.74	52.57 ± 4.45***	44.02 ± 3.22†
**Epididymal fat (g/kg)**	25.08 ± 1.52	23.68 ± 2.77	36.90 ± 2.83**	37.17 ± 3.19
**Total cholesterol (mmol/l)**	1.90 ± 0.25	1.98 ± 0.11	2.00 ± 0.16	1.74 ± 0.13
**LDL (mmol/l)**	0.24 ± 0.03	0.19 ± 0.02	0.40 ± 0.05*	0.22 ± 0.03†
**HDL (mmol/l)**	0.63 ± 0.04	0.65 ± 0.04	0.59 ± 0.04	0.60 ± 0.06
**Triacylglycerols (mmol/l)**	0.90 ± 0.14	0.93 ± 0.16	1.53 ± 0.13**	0.89 ± 0.12††

Data are means ± SEM of 8/10 animals. One and Two-Way ANOVA with Dunnet's and Bonferroni multicomparison test: *p<0.05, **p<0.01, ***p<0.001 *vs* control; †p<0.05, ††p<0.01with *vs* without CSN resection.

ESM Figures

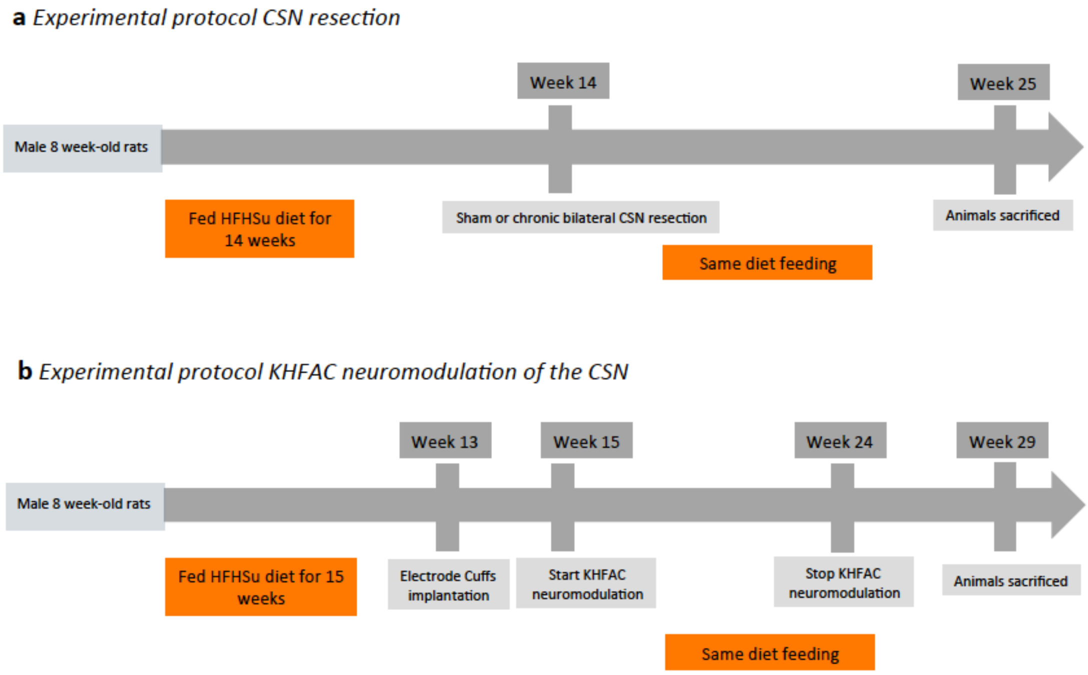

ESM Figure 1 – representative figure of CSN resection protocol (on top) and of CSN KHFAC neuromodulation protocol (bottom).

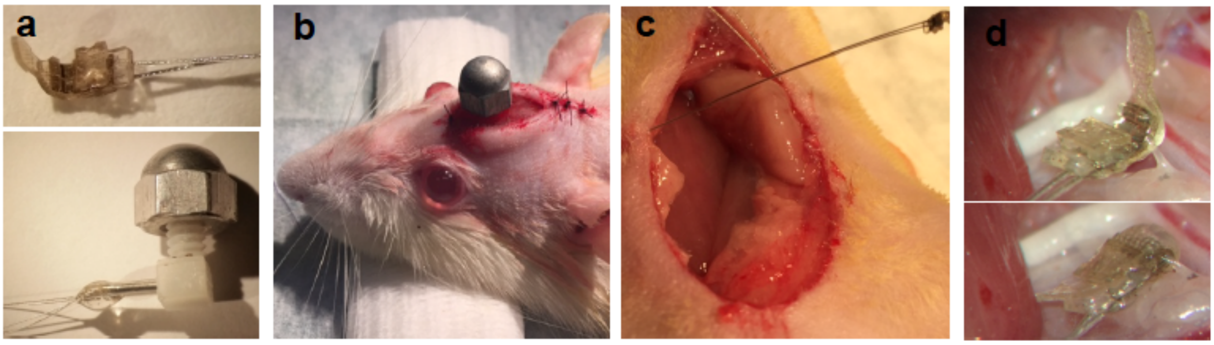

ESM Figure 2 – figure showing implantation of cuffs electrodes. a. shows CorTec cuffs connected to plastic one headcaps; b. shows headcaps cemented in place; c. shows the wires trocated behind the ear to the neck; and d. shows the electrical cuffs placed bilaterally on CSN.

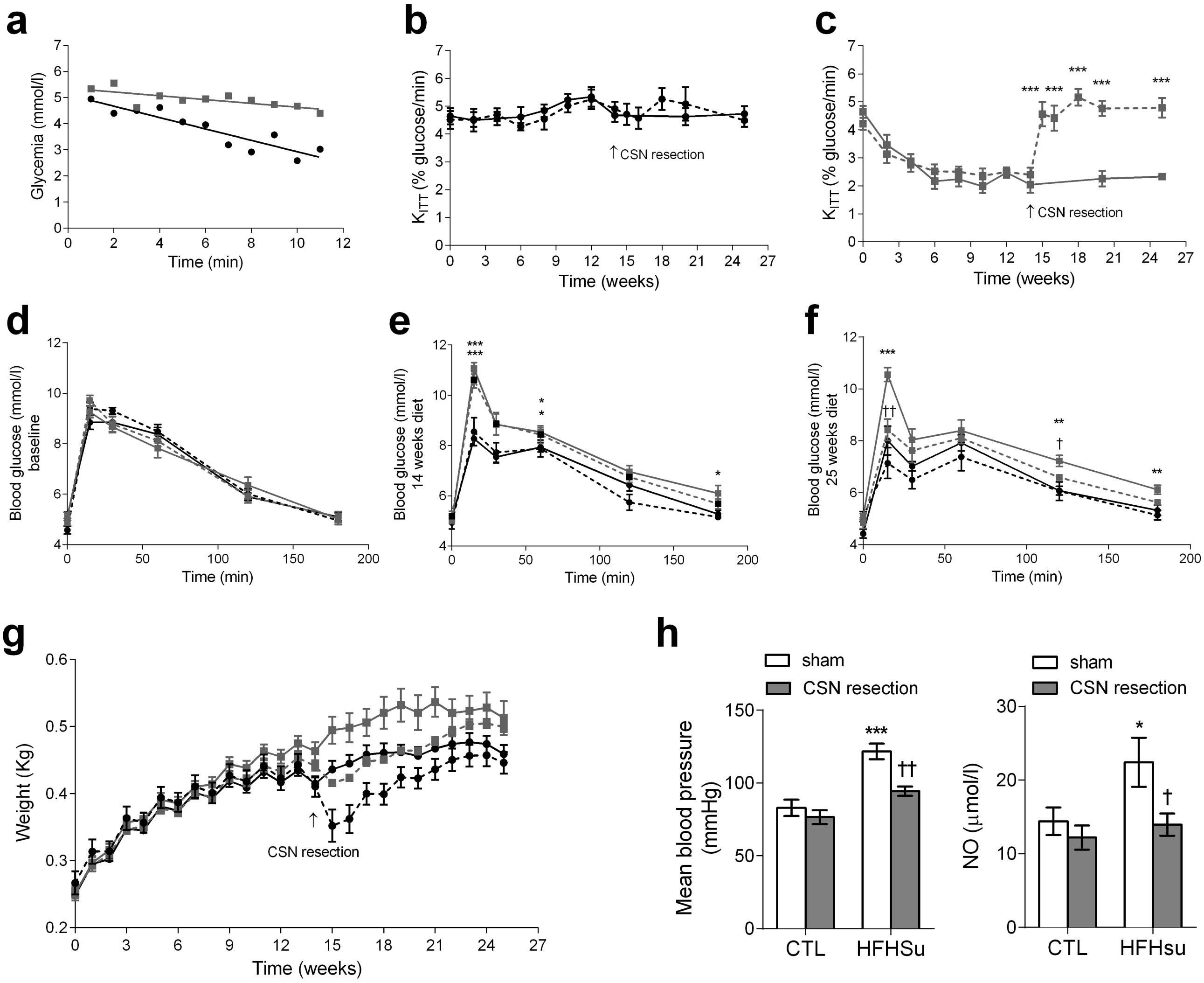

ESM Figure 3 – Effect of chronic carotid sinus nerve (CSN) bilateral resection on cardiometabolic parameters in a high-fat/high-sucrose (HFHSu)-induced type 2 diabetes animal model. **a,** depicted representative glucose excursion curves for insulin tolerance test in a control rat and in the HFHSu animal. **b, c,** shown the effect of CSN resection on insulin sensitivity assessed by an insulin tolerance test and expressed as the constant rate for glucose disappearance (K_{ITT}) in control (b) and HFHSu (c) animals. **d-f,** Effect of CSN resection on glucose tolerance depicted as glucose excursion curves in control and HFHSu animals at baseline (d), before CSN resection (14 weeks of diet) (e) and 11 weeks-post-CSN resection (25 weeks) (f). **g,** Effect of CSN resection on weight gain in control and HFHSu animals. (h) Effect of CSN resection on mean arterial pressure (MAP) and endothelial function and inflammation measured as plasma NO/NO_3^- levels. CSN resection was performed at 14th week of diet and animals were maintained under their respective diets until the 25th week. (a-g)

Black line, control sham; Black dotted line, control with CSN resection; Grey line, HFHSu sham; Grey dotted line, HFHSu with CSN resection. (h) White bars, without CSN resection; grey bars with CSN resection. Data are means ± SEM of 8-10 animals. One and Two-Way ANOVA with Dunnet's and Bonferroni multicomparison test: ** $p<0.01$, *** $p<0.001$ vs control; † $p<0.05$, †† $p<0.01$, with vs without CSN resection.

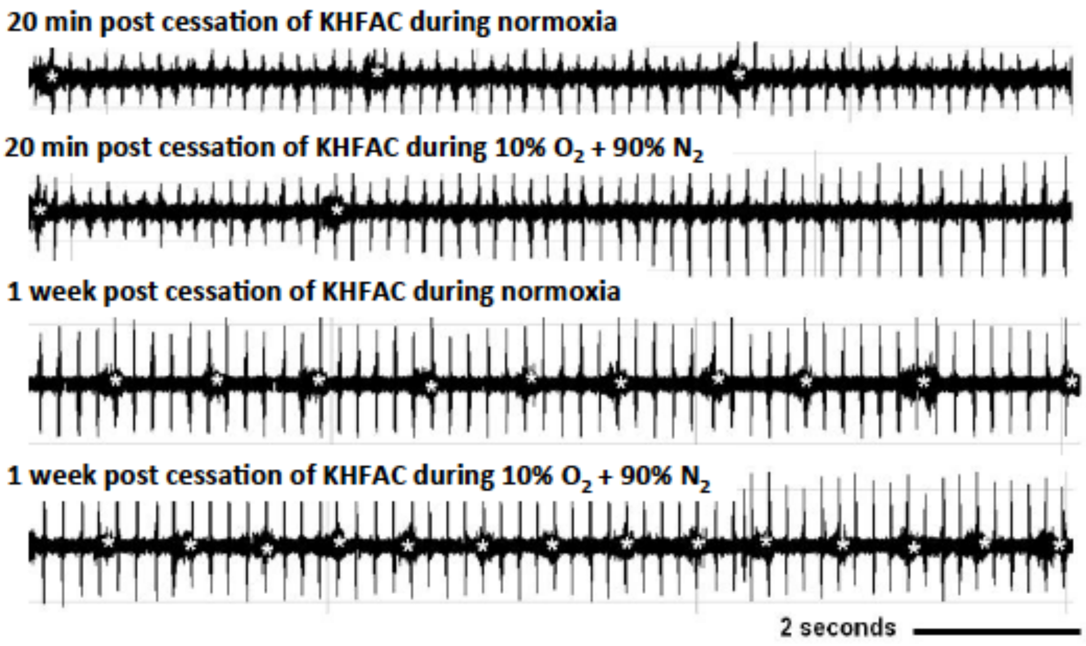

ESM Figure 4 – Effect of one-week of kilohertz alternating frequency current (KHFAC) on EMG recording in a standard-diet animal. Panel shows respectively, from the top to the bottom, EMG recording 20 min after electrical block cessation, in normoxia and hypoxia and EMG recording one-week after block cessation in normoxia and hypoxia. * represents respiratory burst.

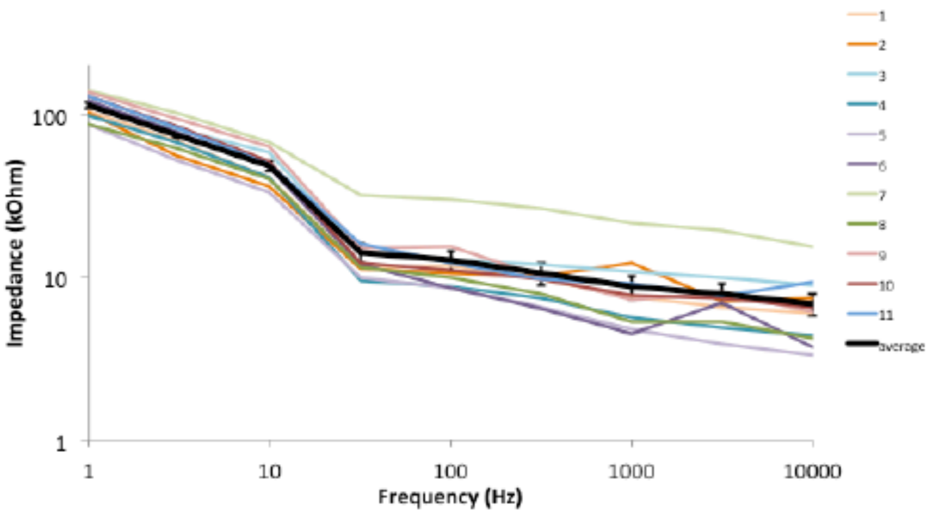

ESM Figure 5 – Electrical impedance of the eleven cuff electrodes implanted bilaterally at the carotid sinus nerve (CSN).

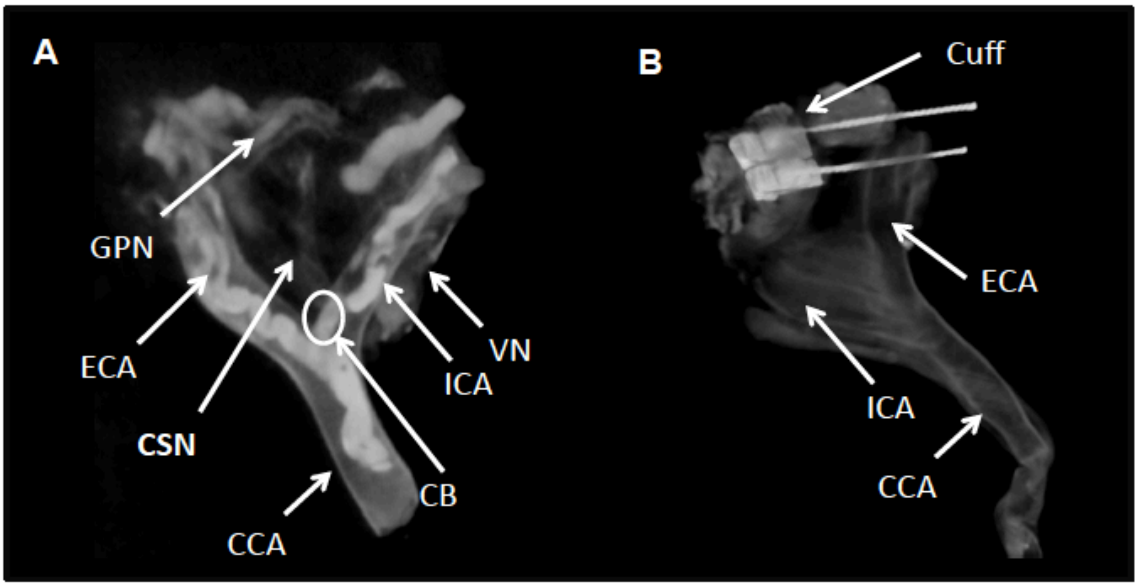

ESM Figure 6 – Micro CT of carotid artery bifurcations with (A) or without (B) electrode cuff implantations at the carotid sinus nerve (CSN). CCA – common carotid artery; ECA – external carotid artery; GPN – glossopharyngeal nerve; ICA – internal carotid artery; VG – vagus nerve.
